# In vitro-activity of oily calcium hydroxide suspension on microorganisms as well as on human alveolar osteoblasts and periodontal ligament fibroblasts

**DOI:** 10.1186/1472-6831-14-9

**Published:** 2014-01-29

**Authors:** Sigrun Eick, Tatjana Strugar, Richard J Miron, Anton Sculean

**Affiliations:** 1 Department of Periodontology, School of Dental Medicine, University of Bern, CH-3010, Bern, Switzerland

**Keywords:** Oily calcium hydroxide suspension, Human alveolar osteoblasts, Periodontal ligament fibroblasts, Periodontopathogens

## Abstract

**Background:**

Findings from animal and human studies have indicated that an oily calcium hydroxide suspension (OCHS) may improve early wound healing in the treatment of periodontitis. Calcium hydroxide as the main component is well known for its antimicrobial activity, however at present the effect of OCHS on the influence of periodontal wound healing/regeneration is still very limited. The purpose of this in vitro study was to investigate the effect of OCHS on periodontopathogenic bacteria as well as on the attachment and proliferation of osteoblasts and periodontal ligament fibroblasts.

**Methods:**

Human alveolar osteoblasts (HAO) and periodontal ligament (PDL) fibroblasts were cultured on 3 concentrations of OCHS (2.5, 5 and 7.5 mg). Adhesion and proliferation were counted up to 48 h and mineralization was assayed after 1 and 2 weeks. Furthermore potential growth inhibitory activity on microorganisms associated with periodontal disease (*e.g. Porphyromonas gingivalis, Tannerella forsythia*, *Aggregatibacter actinomycetemcomitans*) as well as the influence of periodontopathogens and OCHS on the HAO and PDL fibroblasts counts were determined.

**Results:**

More than a 2-fold increase in adherent HAO cells was observed at 4 h following application of OCHS when compared to the control group (p = 0.007 for 2.5 mg). Proliferation of HAO cells at 48 h was stimulated by moderate concentrations (2.5 mg; 5 mg) of OCHS (each p < 0.001), whereas a high concentration (7.5 mg) of OCHS was inhibitory (p = 0.009). Mineralization was observed only for HAO cells treated with OCHS. OCHS did not exert any positive effect on attachment or proliferation of PDL fibroblasts. Although OCHS did not have an antibacterial effect, it did positively influence attachment and proliferation of HAO cells and PDL fibroblasts in the presence of periodontopathogens.

**Conclusions:**

The present data suggests that OCHS promotes osteoblast attachment, proliferation and mineralization in a concentration-dependent manner and results are maintained in the presence of periodontal pathogens.

## Background

Periodontitis is a bacterially induced chronic inflammatory disease, and an imbalance of innate immune-defence system markedly contributes to the destruction of the periodontium [1]. A small group of predominantly gram-negative anaerobic or microaerophilic bacteria within plaque is associated with initiation and progression of periodontitis. Organisms strongly implicated as etiologic agents of periodontitis include *Aggregatibacter actinomycetemcomitans*, *Porphyromonas gingivalis*, *Tannerella forsythia* and *Treponema denticola*[2]. Moreover, other species such as *Campylobacter rectus*, *Eubacterium nodatum*, *Fusobacterium nucleatum*, *Prevotella intermedia*, *Parvimonas micra, Streptococcus constellatus* support pathogenesis of disease [2,3]. Bacteria interact with host cells resulting in expression of inflammatory mediators, transition of polymorphonuclear neutrophils to the gingival crevice [1,4]. Host response contributes to tissue destruction and bone resorption with the main mechanism of the ratio of RANKL (receptor-activator of nuclear-factor-κB ligand) to osteoprotegerin [1].

It has been well documented that resolution of inflammation and stop of disease progression can be predictably obtained with nonsurgical and conventional surgical periodontal therapy [5]. The ultimate goal of periodontal therapy is however the regeneration of the tooth’s supporting structures lost due to periodontal disease and should result in formation of new root cementum, periodontal ligament and bone [6]. Treatment with barrier membranes alone or in combination with different grafting materials, the use of biologic active substances such as enamel matrix proteins or growth factors have been shown to promote periodontal regeneration and to significantly improve the clinical outcomes evidenced by probing depth reduction, clinical attachment gain and defect fill [7]. A few of the materials have been described to act antimicrobial, *e.g*., enamel matrix derivatives inhibit the growth of *P. gingivalis*[8]. A study analysing the influence of five different biomaterials used for regenerative periodontal surgery against two *A. actinomycetemcomitans* showed antimicrobial activity of two materials against one of the two tested strains, most inhibitory was an oily calcium hydroxide suspension [9].

This oily calcium hydroxide containing paste (OCHS) (Osteora®, previously Osteoinductal®, DFS-Diamon GmbH Riedenburg, Germany) has been suggested to possess properties which may positively affect periodontal wound healing/regeneration [10-14]. It is based on calcium hydroxide (Ca(OH)_2_) and uses a carrier substance consisting of synthetically produced porcine oleum pedum and vaselinum album. Calcium hydroxide, a white odourless powder with a low solubility in water, has antibacterial properties by the release of highly reactive hydroxyl ions in aqueous fluids which damages cytoplasmatic membranes, proteins and DNA [15]. In endodontic treatment it is used as a pulp-capping agent [16], as a disinfectant for root canal treatment [17] and for apexification after pulp death [18]. In the pulp, a superficial necrosis induced by the high pH occurs with a mild inflammatory response and hard tissue formation in the environment [19].

Several animal studies which have evaluated the effects of OCHS on bone regeneration in various types of defects yielded different outcomes [10,11,20,21]. In a guided bone regeneration model using minipig calvaria, OCHS failed to exert osteoinductive properties and hampered bone healing when used in conjunction with guided bone regeneration [22]. Furthermore, the healing of endosseous implants was not improved when those were inserted together with OCHS [21]. On the other hand, application of OCHS during the osteotomy phase of distraction osteogenesis improved formation of new bone [10] while in experimentally created intrabony periodontal defects, the application of OCHS in conjunction with access flap surgery promoted periodontal regeneration [11].

Varying outcomes related to wound healing and regeneration were also found in the few clinical studies. In one study, OCHS improved early wound healing when used in conjunction with non-surgical therapy [12]. In another controlled clinical trial evaluating the healing of intrabony defects treated with access flap surgery with and without OCHS, significantly higher pocket depths reductions and clinical attachment level gains were found in the defects treated filled with OCHS compared to the controls (i.e. access flap surgery alone) [13]. On the contrary, another recent controlled clinical study using a similar design has failed to demonstrate any superior outcomes following the application of OCHS when compared with access flap surgery alone [23].

Although much investigation has been obtained in animal and clinical models, knowledge about its mode of action and effects on periodontal ligament (PDL) cells, bone-forming osteoblasts as well as oral microbes is still limited. The aim of the present study was two-fold; 1) To determine a potential antimicrobial activity of OCHS including its components against bacterial species involved in pathogenesis of periodontitis and 2) to determine the effect on attachment and proliferation of host cells (periodontal ligament fibroblasts and osteoblasts).

## Methods

### Test substances

OCHS (Osteora®, DFS-DIAMON GmbH, Riedenburg, Germany) was used. According to manufacturer’s information it is composed of 20% w/w Ca(OH)_2_, 40% oleum pedum and 40% vaselinum album. In the experimental design the use of OCHS itself as well as Ca(OH)_2_ and oleum pedum were used as test substances.

#### Determination of antimicrobial efficacy of oily calcium hydroxide suspension

The following species have been tested in the antimicrobial assays: *F. nucleatum* ATCC 25586, *P. intermedia* ATCC 25611, *P. gingivalis* (ATCC 33277 and three clinical isolates), *T. forsythia* ATCC 43037, *A. actinomycetemcomitans* (Y4 and three clinical isolates), *C. rectus* ATCC 33238, *Eikenella corrodens* ATCC 23834, *E. nodatum* ATCC 33099, *P. micra* ATCC 33270, and *Capnocytophaga gingivalis* ATCC 33624. All strains were precultivated at 37°C in appropriate conditions (anaerobic except for *A. actinomycetemcomitans* – 5% CO_2_) 42 h before experiments. Modified tryptic soy agar [24] was used as cultivation media.

First, micro-broth dilution technique was used to determine the MICs. After subcultivation of bacterial strains, a defined inoculum (McFarland 0.5) was added in a ratio of 1 : 9 to broth containing the test substances. Oleum-pedum (solubilized with Tween 20 in a ratio 1 : 1) was tested in a concentration of 0.625% - 20%, Ca(OH)_2_ in a concentration of 3.13 mg/ml - 100 mg/ml adapted to the concentration available in OCHS. In experiments testing oleum pedum, each test tube contained 20% Tween 20. After an incubation time of 42 h (18 h aerobes), the growth of microbes was analyzed by visual checking of turbidity and subcultivation. For OCHS as solvent agents DMSO, Tween 20 and different oils have been proven but it was impossible to solubilize OCHS. Therefore, the standard methods for determination of antimicrobial activity against anaerobes and other slow-growing microorganisms (microbroth-dilution, agardilution) were not applicable. Finally a modified agar diffusion method was used as follows: One hundred μl of bacterial suspension (MacFarland 0.5) were spread on agar plates (Wilkins Chalgren agar supplemented with 5% blood). Then, each two gaps were prepared using a cork borer (diameter 7 mm). After that, the gap was filled with 100 μL of agar followed by the test substance (50 mg and 100 mg of OCHS). After incubation at 37°C in the anaerobic atmosphere for 42 h, the inhibition zones were measured.

To exclude a growth-promoting effect of OCHS, suspensions of selected bacterial strains (*P. gingivalis* ATCC 33277, *P. gingivalis* M5-1-2, *A. actinomycetemcomitans* Y4 and *F. nucleatum* ATCC 255866) were added to 200 μL nutrient broth added with 50 mg of OCHS (20%w/v). Tubes had been incubated for 24 h anaerobically. Immediately before removing 25 μl of mixture, suspensions were mixed by vortexing and short centrifugation at 400 *g*. The removed 25 μl were serially diluted and each 100 μl were plated on agar plates. The numbers of viable bacteria were determined by counting the colony forming units (cfu).

### Effect of oily calcium hydroxide suspension on osteoblasts and periodontal ligament fibroblasts

In the second part of the study, human alveolar osteoblasts (HAO) as well as human PDL fibroblasts were used. Both cell types were obtained from three periodontally healthy patients during surgery (extraction of teeth for orthodontic reasons). Human bone chips were cultured from an explant model as previously described [25,26]. Following collagenase digestion, HAO cells were plated in T-75 flasks containing cell cultivation medium (DMEM, Invitrogen, Carlsbad, CA) supplemented with 10% of fetal bovine serum (FBS, Invitrogen). PDL cells were harvested from the middle third portion of tooth and placed in T-25 cell culture flasks till cell confluency [27]. The cultivation medium was DMEM supplemented with 10% FBS. For experiments, both cell types were used from passages 4–6. The identity of the cells was confirmed as described recently [26,27]. Using the tissue for research purposes was approved by the Ethical commission of the Canton Bern. All patients gave their consent.

In the experiments, slides were placed into 24-well plates and covered with the test substances. The test substances were OCHS in three different concentrations (1 U, 2 U, 3 U). 1 U represented 2.5 mg of total material (meaning 2 U is equivalent to 5 mg and 3 U to 7.5 mg). In addition, calcium hydroxide in aqueous solution (1.5 mg corresponding to 3 U of OCHS) and oleum pedum substance (3 mg corresponding to 3 U of OCHS) were used. Uncovered slides served as negative controls. Immediately thereafter, HAO cells were added at a density of 10,000 cells/well and the wells were incubated at 37°C with 5% CO_2_. Cells were fixed and stained for adhesion and proliferation experiments at 2 h, 4 h, 24 and 48 h using DAPI staining. Cell differentiation was analyzed by determination of alkaline phosphatase activity and mineralization by using 2% alizarin red S staining 1 and 2 weeks post-seeding. Each 10 fields of 1 mm^2^ were counted. Fields were selected equally distributed from the whole slide. A counting grid was used and each subfield (50 μm × 50 μm) with positive staining for calcium noduli was counted in relation to the total numbers of subfields; the mean was used as a single value for analysis. The mineralization was measured 1 and 2 weeks after beginning the experiments. To ensure that only mineralization of cells was counted, OCHS with no cells was used as a negative control.

Similarly to HAO cells, effects on PDL fibroblasts were determined. Slides which have been placed into 24-well plates were covered with the test substances. PDL fibroblasts were added at a density of 10,000 cells/well. Cells were fixed and stained for adhesion and for proliferation experiments at 2 h, 4 h, 24 h and 48 h using DAPI staining as described for HAO cells.

#### Determination of the effect of oily calcium hydroxide suspension on PDL fibroblasts and osteoblasts interaction with microorganisms

The concentration of 2 U (5 mg) OCHS was selected and placed on each well of a 24-well plate for experiments focusing on the interaction of host cells with bacterial strains.

HAO cells and PDL fibroblasts respectively were seeded on the slides with and without coverage of 2 U of OCHS. *A. actinomycetemcomitans* Y4 as well as the combination of *P. gingivalis* ATCC 33277, *T. forsythia* ATCC 43037 and *T. denticola* ATCC 35405 were added. The bacterial load was always 10^6^ per well. The HAO cells and PDL fibroblasts respectively were fixed and stained at 4 h.

### Statistical analysis

Except for the antimicrobial assays (independent replicates) at least six independent experiments were made per group. More than two independent groups were compared by one way ANOVA followed by Post Hoc LSD analysis for comparison with the controls (activity of OCHS and its components on HAO cells and PDL fibroblasts). Statistical analysis was made by using Student’s t-test for two independent samples (effect of OCHS on interaction HAO cells’ and PDL fibroblasts’ interaction with bacteria).

## Results

### Oily calcium hydroxide suspension does not act antibacterial

The MIC values of oleum pedum and calcium hydroxide (the main components of OCHS) are presented in Table 1. Whereas oleum pedum did not exert any antibacterial effect, Ca(OH)_2_ was inhibitory; the MIC values were in the range of 6.25 – 25 mg/ml.

**Table 1:** Minimal inhibitory concentrations of the components of OCHS (porcine oleum pedum and calcium hydroxide) determined by micro-broth dilution technique

	**Porcine oleum-pedum**	**Ca(OH)**_**2**_
*F. nucleatum* ATCC 25586	> 20%	6.25 mg/ml
*P. intermedia* ATCC 25611	> 20%	6.25 mg/ml
*P. gingivalis* ATCC 33277	> 20%	12.5 mg/ml
*P. gingivalis* M5-1-2	> 20%	12.5 mg/ml
*P. gingivalis* J430-1	> 20%	25 mg/ml
*P. gingivalis* MaRL	> 20%	25 mg/ml
*T. forsythia* ATCC 43037	> 20%	12.5 mg/ml
*A. actinomycetemcomitans* Y4	> 20%	12.5 mg/ml
*A. actinomycetemcomitans* J1	> 20%	12.5 mg/ml
*A. actinomycetemcomitans* J2	> 20%	12.5 mg/ml
*A. actinomycetemcomitans* J7	> 20%	25 mg/ml
*C. rectus* ATCC 33238	> 20%	12.5 mg/ml
*E. corrodens* ATCC 23834	> 20%	50 mg/ml
*E. nodatum* ATCC 33099	> 20%	12.5 mg/ml
*P. micra* ATCC 33270	> 20%	25 mg/ml
*C. gingivalis* ATCC 33624	> 20%	12.5 mg/ml

As mentioned in the materials and methods, due to the insolubility of OCHS, a modified agar diffusion technique was used to determine a possible antimicrobial effect of OCHS. These experiments however did not reveal any inhibition zone by OCHS in the two tested concentrations. The final experiments cultivating bacterial suspensions in nutrient broth underlined that OCHS does not influence growth of periodontopathogens in any way. Neither growth-suppressing nor growth-promoting effects were visible; differences to controls were always below 0.2 log_10_ cfu (data not shown). It has to be noted that due to the insolubility of OCHS, the tubes contained two layers; one of OCHS and one of broth. Thus only at the interface compounds released from OCHS might interfere with bacteria.

### Oily calcium hydroxide suspension may promote adhesion of osteoblasts and mineralization but does not affect adhesion and proliferation of PDL fibroblasts

**Figure 1:**
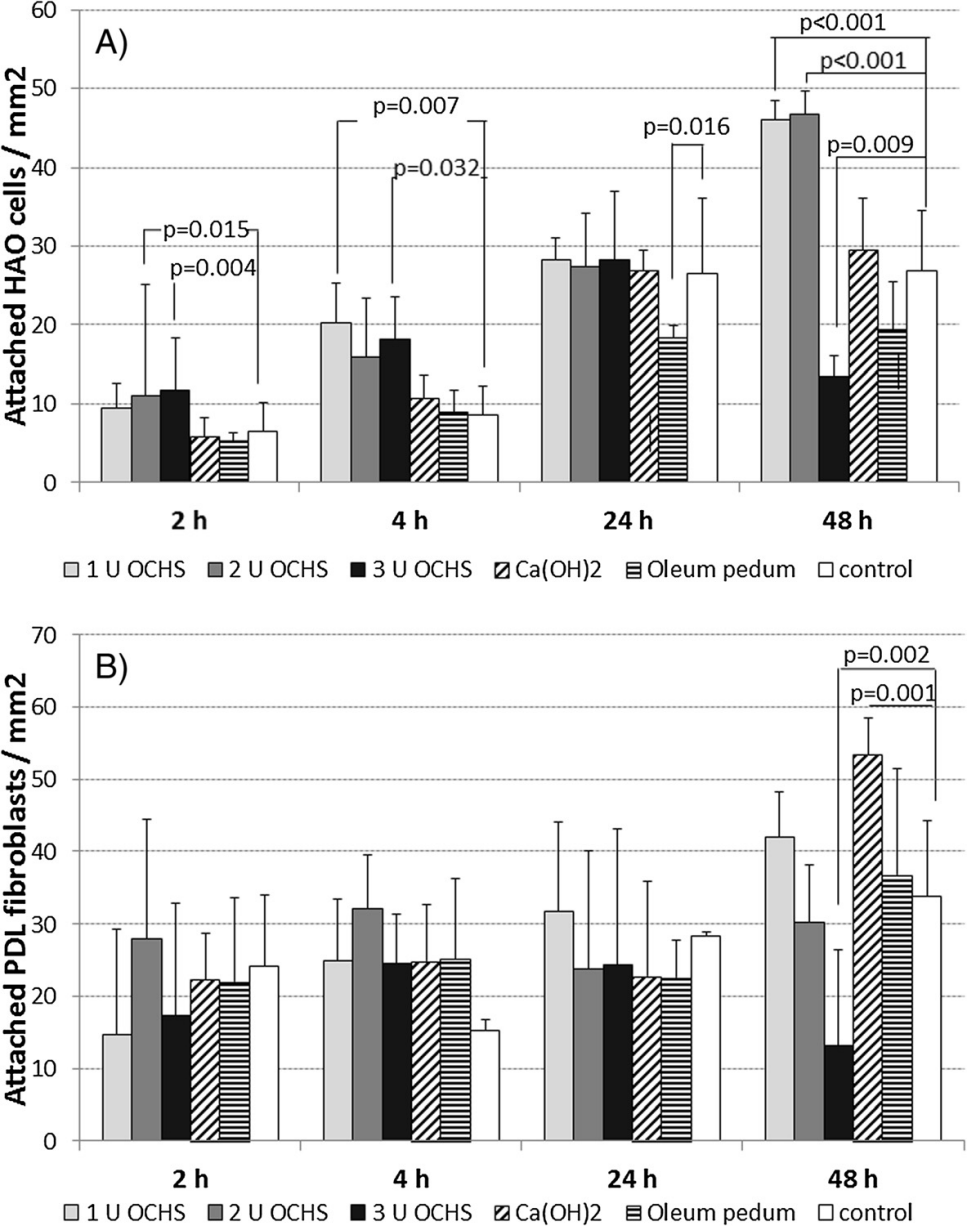
**Attachment and proliferation of A) HAO cells and B) PDL fibroblasts (mean and SD) each after coverage with different amounts of oily calcium hydroxide suspension (OCHS) as well as 1.5 mg Ca(OH)**_**2 **_**and 3.0 mg oleum pedum (p-values in comparison with controls were determined by Post Hoc LSD analysis after ANOVA).**

Addition of OCHS promoted the attachment of HAO cells. After 2 h, 11.00 ± 4.90 cells/mm^2^ adhered to a surface covered with 2 U of OCHS. Using 3 U the value was 11.67 ± 2.89 cells/mm^2^. These differences were significant in comparison to the control, where 6.57 ± 3.55 HAO cells were found per mm^2^ on the surface. After 4 h, 20.33 ± 14.13 HAO cells/mm^2^ were counted after coverage with 1 U of OCHS and 18.11 ± 6.66 HAO cells/mm^2^ when 3 U of OCHS were used being significantly different from the controls (8.67 ± 3.50 HAO cells/mm^2^). Stimulated proliferation was found 48 h after starting the experiments and coverage with 1 U and 2 U of OCHS (1 U: 46.00 ± 13.11 HAO cells/mm^2^, 2 U: 46.67 ± 18.23 HAO cells/mm^2^ in comparison to control: 26.89 ± 7.25 HAO cells/mm^2^). In contrast to the 1 U and 2 U of OCHS, the high concentration of 3 U significantly inhibited the proliferation of HAO cells (13.44 ± 5.20 HAO cells/mm^2^). An influence of the used Ca(OH)_2_ concentration was not registered. Coverage with oleum pedum was followed by a decreased cell number 24 h after addition of the cells. The results including significant values are presented in Figure 1.

**Figure 2:**
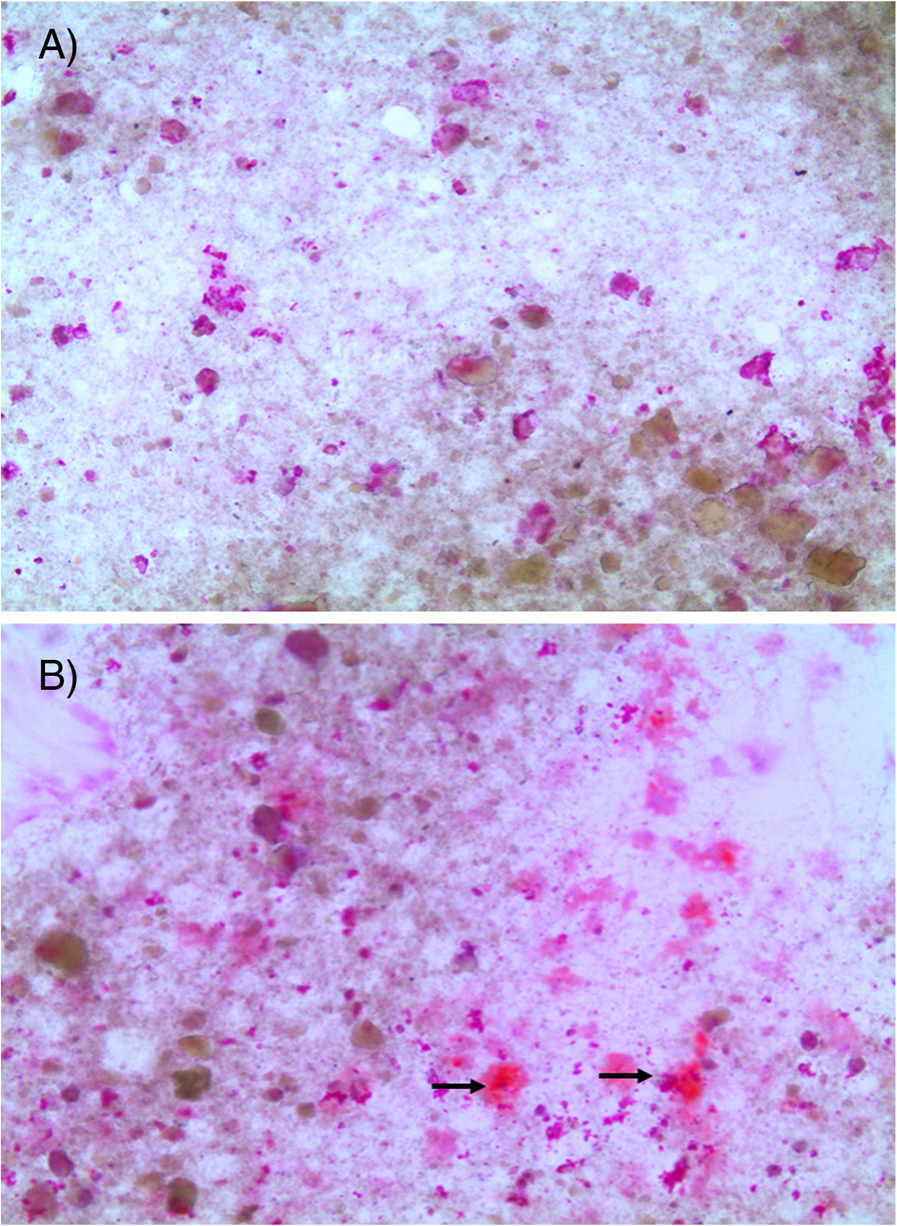
**Cell mineralization stained with alizarin red one week after seeding HAO cells.** The mineralization is visible by the pink color (→), **A)** untreated HAO cells, **B)** HAO cells seeded on OCHS.

The differentiation and mineralization of the HAO cells was also analyzed. In all experiments only HAO cells positively stained for alkaline phosphatase were found. Without addition of OCHS, no mineralization was present on the surface. When the surface was covered with OCHS, extracellular mineralization of HAO was detectable after one week (5.57 ± 1.97% stained area; Figure 2). The quantity did not significantly change after two weeks (4.12 ± 1.59%).

Addition of OCHS did not have any significant influence on attachment of PDL fibroblasts. Reduced proliferation was found 48 h after starting the experiments and coverage with 3 U of OCHS (13.22 ± 4.12 PDL fibroblasts/mm^2^) in comparison with the controls (33.89 ± 17.48 PDL fibroblasts/mm^2^). In contrast, Ca(OH)_2_ stimulated the proliferation of PDL fibroblasts (53.44 ± 15.22 PDL fibroblasts/mm^2^ compared to control values at the 48 h time-point (Figure 1).

The results suggested a possible cytotoxic effect. Therefore, HAO cells and PDL fibroblasts were seeded on slides covered with the test substances as described above. Cells were incubated for 4 h and 48 h. Thereafter the percentage of viable cells was determined by trypan blue exclusion test.

**Figure 3:**
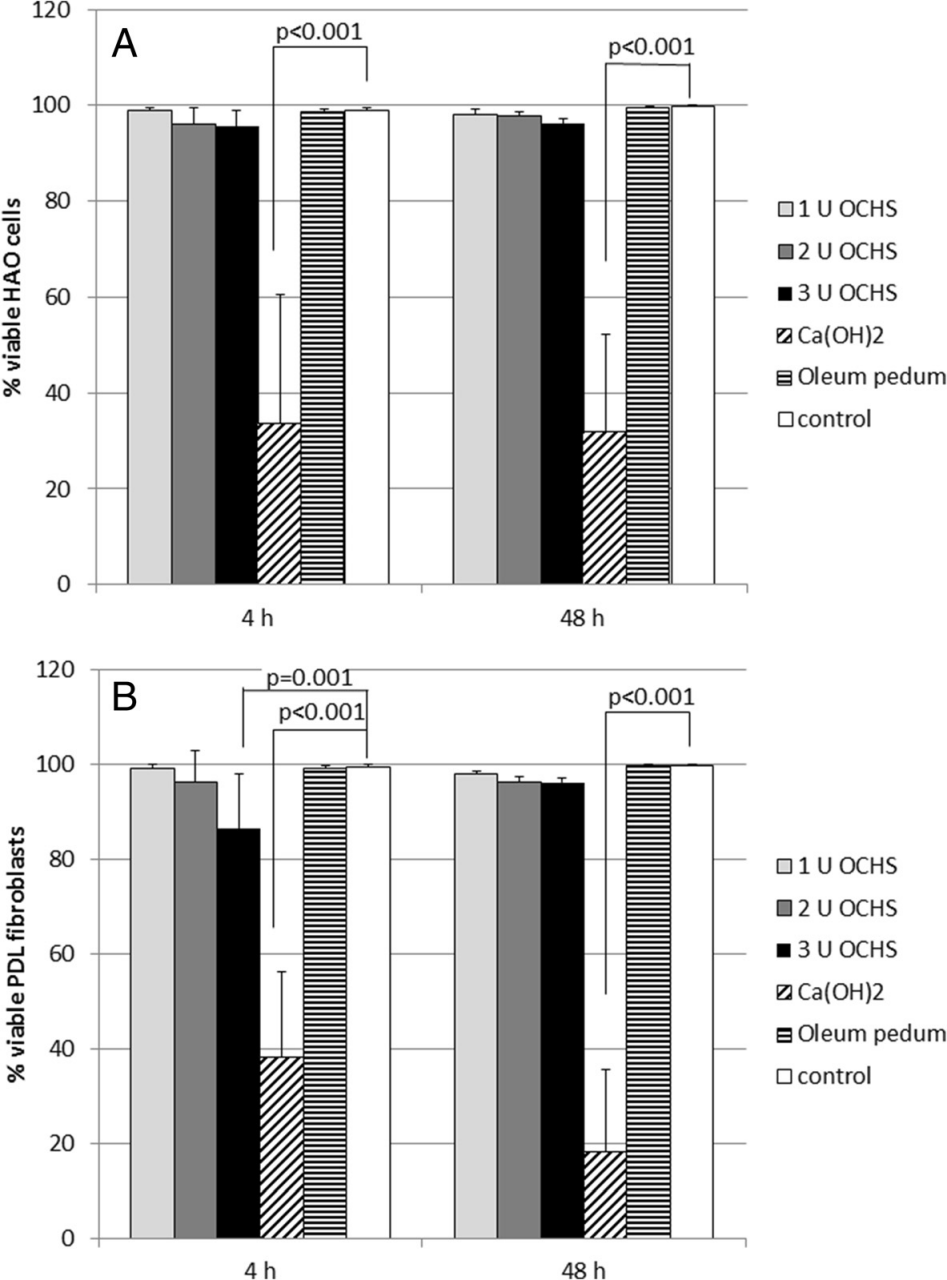
**Percentage of viable A) HAO cells and B) PDL fibroblasts (mean and SD) after coverage with different amounts of oily calcium hydroxide suspension (OCHS) as well as 1.5 mg Ca(OH)**_**2 **_**and 3.0 mg oleum pedum determined by trypan exclusion test (p-values in comparison with controls were determined by Post Hoc LSD analysis after ANOVA).**

A remarkable higher percentage of dead cells was always found after pretreatment with 1.5 mg Ca(OH)_2_ in comparison with controls (each p < 0.001). The viability of the cells determined by trypan exclusion test changed slightly after pretreatment with OCHS, the difference to untreated controls was significant for PDL fibroblasts 4 h after starting experiments (Figure 3).

### Bacteria do not interfere with the effect of oily calcium hydroxide suspension on adhesion and proliferation of osteoblasts and PDL fibroblasts

**Figure 4:**
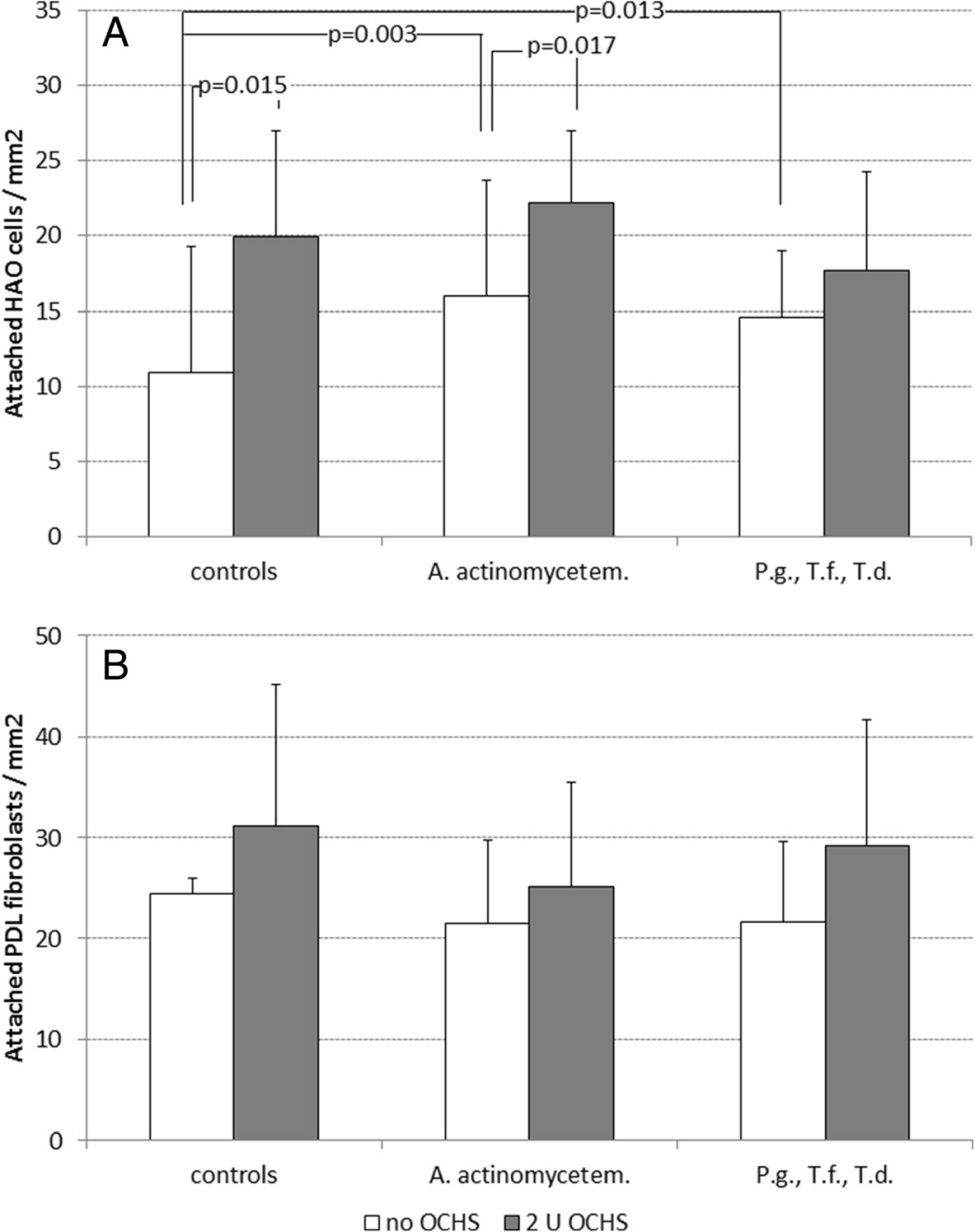
**Attachment of A) HAO cells and B) PDL fibroblasts (mean and SD) 4 h after coverage with and without oily calcium hydroxide suspension (OCHS) and addition of *A. actinomycetemcomitans *** **Y4 as well as the combination of *P. gingivalis *** **ATCC 33277, *T. forsythia *** **ATCC 43037, *T. denticola *** **ATCC 35405 (p-values in comparison with controls and with no OCHS respectively each were determined by Student’s t-test).**

The addition of bacteria did not significantly change the numbers of adhered HAO cells when the slides were covered with 2 U of OCHS. If *A. actinomycetemcomitans* Y4 was present, an increase of HAO cells with 2 U of OCHS (25.08 ± 10.04 HAO cells/mm^2^) was still significant in comparison with those without OCHS (controls). Surprisingly, the addition of bacteria enhanced the numbers of attached HAO cells (*A. actinomycetemcomitans* Y4: 16.00 ± 4.44 HAO cells/mm^2^, *P. gingivalis*, *T. forsythia*, *T. denticola* in mixture: 14.56 ± 4.07 HAO cells/mm^2^) being significantly higher than the controls (10.87 ± 8.43 HAO cells/mm^2^). Contact with bacteria did not significantly influence the numbers of attached PDL fibroblasts (Figure 4).

## Discussion and conclusions

OCHS is an oily suspension which contains Ca(OH)_2_ as the active constituent. Other components are the synthetically produced oleum pedum tauri, a carrier material containing triglycerides including oil acid, palmitin acid, hexadecen acid and vaselinum album.

An antibacterial effect by OCHS was not observed even in extremely high concentrations by performing a modified agar diffusion test. Only one previous study reported on the effects of OCHS on periodontopathogens. In that study, OCHS acted inhibitory on *A. actinomycetemcomitans* strain ATCC 33384 (serotype c), however no antibacterial effect was observed following culture of *A. actinomycetemcomitans* strain ATCC 43718 (serotype b) with OCHS [9]. We also included in our study one clinical isolate belonging to serotype c. In contrast to the results described previously [9], we did not observe any antibacterial effect following application with OCHS in the present study on either tested strains of *A. actinomycetemcomitans*.

Due to the insolubility of the material, we were only able to determine the minimal inhibitory concentrations of individual components of OCHS by a micro-broth dilution technique. It was observed that although oleum pedum did not have any antibacterial property, the use of calcium hydroxide in high concentrations such as those present in OCHS acted as growth-inhibitory. Calcium hydroxide is used widely in endodontic treatment and in vitro evidence demonstrates it suppresses the growth of *Candida albicans*[28] and certain other anaerobically growing bacteria [29] although limited antimicrobial efficacy has translated in vivo following the analysis of root canals post-treatment [30,31]. In our in vitro assays, in contrast to calcium hydroxide, OCHS did not have any antibacterial effect which may suggest a counteracting effect of the other components of OCHS to calcium hydroxide. Due to the nature of periodontal therapy, bacteria associated with periodontitis should be eliminated or reduced in any treatment modality. The missing antimicrobial activity of OCHS suggests the additional use of an antimicrobial, such as chlorhexidine. For these reasons, addition of 0.4% CHX to a calcium hydroxide paste did not affect osteoblastic cell biology in vivo [32].

In the present study, we investigated the effects of OCHS on HAO on cell attachment and proliferation. Our results demonstrated increased cell behavior following culture with OCHS and demonstrated concentration sensitivity. Low to moderate concentrations of OCHS acted clearly stimulatory, whereas the application with a concentration of 3U (7.5 mg) demonstrated in part detrimental results. To retard the release of Ca(OH)_2_, OCHS contains a high percentage of oleum pedum. We investigated HAO counts cultured in the presence of oleum pedum and found a reduction in cell behavior follwing its application. Oleum pedum seems to counteract the stimulating activity of Ca(OH)_2._ In an animal model using open Teflon capsules, OCHS inhibited bone formation and an active resorption of OCHS was not observed [20]. It can be suggested that the missing degradable properties are certainly from the oleum pedum and a more pronounced effect appears to be observed with increasing concentrations. A second logical explanation might be due to the cytotoxicity of OCHS. Although oleum pedum prevents the cytotoxicity of Ca(OH)_2_, toxic effects to a certain degree were seen when OCHS was used at high concentrations. This obvious sensitivity of the used concentration might partly explain the reported different outcomes in animal and clinical studies. Accordingly, following our first results sets of experiments, a moderate concentration was chosen for ongoing experiments based on its positive results. Using this concentration, an increase in osteoblast mineralization was found 1 – 2 weeks post seeding with culture media containing OCHS. Recently it was demonstrated that Ca(OH)_2_ is able to stimulate mRNA expression of bone sialoprotein and Runx2 [33]. Runx2 is an essential transcription factor in osteoblast differentiation [34] and bone sialoprotein is a late marker for osteoblast differentiation found during the mineralization process of osteoblasts [35].

Despite the stimulatory effects of OCHS on osteoblasts, OCHS did not exert any positive effect on attachment or proliferation of PDL fibroblasts. Similarly to our HAO experiments, the highest used concentration of OCHS also reduced proliferation of PDL fibroblasts after 48 h. Proliferation of PDL fibroblasts was induced by Ca(OH)_2_ as described recently; but in contrast to our results, OCHS also had a stimulatory effect on proliferation in that study [36]. In a similar study [14] these authors found high attachment of PDL fibroblasts on OCHS treated root surfaces. Future research to determine the mechanisms influencing the attachment of cells via integrin binding and its downstream molecular mechanisms in host cells (*e.g*. cellular pathways) should investigated.

Following experimental testing of OCHS with cells from the periodontium, a co-culture system was designed to determine the influence of OCHS on cells exposed to periodontal pathogens simultaneously to simulate a clinical setting. Exposure of HAO cells and PDL fibroblasts to periodontopathogenic bacteria did not negatively influence the promoting effect of OCHS on attachment and proliferation of these cells. Therefore, the results from this experiment demonstrate the potential use of OCHS even in the presence of periodontal pathogens without affecting the potential benefit from OCHS. Surprisingly, the addition of bacteria enhanced the number of attached HAO cells. This result should be taken with caution and might be associated with the in vitro culture conditions. The effect was especially pronounced when *A. actinomycetemcomitans* was used. The cytotoxic potential of periodontopathogens is well known. Gingipains are responsible for the majority of the proteolytic activity of *P. gingivalis*[37] and are able to inhibit proliferation of osteoblasts by causing early G1 arrest in cell cycle [38]. A capsular-like polysaccharide antigen of *A. actinomycetemcomitans* induces apoptotic cell-death in osteoblastic cells [39]. But recently it was shown that a low concentration of *A. actinomycetemcomitans* LPS in contrast to high concentrations was able to increase bone sialoprotein gene transcription [40]. A topic of future research might be the interfering and interaction of periodontopathogens with host cells and regenerative materials.

Although, our in vitro studies did not consider the complexity of an in vivo system, the results from the present study may partly explain the different outcomes observed in animal and clinical studies following use with OCHS. In conclusion, OCHS promotes attachment and proliferation of osteoblasts as well as mineralization of their tissue. The effects seem to be concentration-dependent and too high a concentration acts negatively, thus suggesting an application of a moderate concentration. Infection of PDL fibroblasts and osteoblasts with certain periodontopathogens did not negatively interfere with the attachment and early proliferation of osteoblasts and PDL fibroblasts. Nevertheless, the missing antimicrobial activity of OCHS suggests an additional use of an antimicrobial for clinical application. Further in vitro studies should be aimed to investigate the cellular pathways and mechanisms by which these interactions take place.

## Abbreviations

DAPI: 4’,6-Diamidino-2-phenylindole dihydrochloride; DMSO: Dimethyl sulfoxide; FBS: fetale bovine serum; HAO: Human alveolar osteoblasts; MIC: Mimimal inhibitory concentration; OHCS: Oily calcium hydroxide suspension; PDL: Periodontal ligament.

## Competing interests

The authors declare that they have no competing interests.

## Authors’ contribution

SE, RM and AS participated in planning and designing the study, in the data analysis and drafting of the manuscript. TS performed most of the laboratory work and participated in the data analysis as well as drafting the manuscript. All authors have read and approved the final manuscript.

## Pre-publication history

The pre-publication history for this paper can be accessed here:

http://www.biomedcentral.com/1472-6831/14/9/prepub
